# Evaluating the efficacy of multi-incision and tube-dragging therapy combined with laser closure for high horseshoe-shaped anal fistula: Protocol of a prospective, randomized, controlled trial

**DOI:** 10.1371/journal.pone.0307653

**Published:** 2024-09-27

**Authors:** Min Yang, Zubing Mei, Qingming Wang, Ye Han, De Zheng

**Affiliations:** 1 Department of Anorectal Surgery, Shuguang Hospital, Shanghai University of Traditional Chinese Medicine, Shanghai, China; 2 Anorectal Disease Institute of Shuguang Hospital, Shanghai, China; PLOS: Public Library of Science, UNITED KINGDOM OF GREAT BRITAIN AND NORTHERN IRELAND

## Abstract

**Introduction:**

High horseshoe-shaped anal fistula (HHAF) is a complicated and challenging condition that presents considerable obstacles in treatment. We are presently investigating a novel surgical technique involving a combination of multi-incision and tube-dragging therapy, and laser closure (MITD-LaC) for the management of HHAF. Due to the current scarcity of rigorous evidence evaluating this approach, it is essential to perform a well-designed randomized controlled trial to compare the effectiveness of this new method with incision and thread-drawing therapy.

**Methods and analysis:**

This trial is a prospective, randomized, controlled and interventional study. After preliminary screening of qualified outpatients, a total of 64 adult patients will be enrolled in the trial and randomly allocated to either the MITD-LaC group or the control group (n = 32 per group). These patients will receive either MITD-LaC or incision and thread-drawing therapy. The design aims to allow for a robust comparison between the two treatment modalities. The primary endpoint is the wound healing time, while secondary endpoints include postoperative anal pain at 1, 3, and 5 days (measured with visual analogue scale), fecal incontinence score within 30 days after operation (measured with Cleveland Clinic Florida incontinence score), and the occurrence of postoperative complications within 1 month after surgery, and quality of life up to six months postoperatively (evaluated by The Quality of Life in patients with Anal Fistula Questionnaire Score).

**Discussion:**

This study represents the first randomized controlled trial evaluating the short-term outcomes of MITD-LaC, thereby aiming to contribute high-quality evidence to guide clinical practice. Moreover, this trial incorporates comprehensive outcome measures assessing both subjective and objective dimensions. Because of this multidimensional assessment, MITD-LaC offers a promising potential for broader application in the treatment of HHAF. Consequently, obtaining more definitive and authoritative evidence through scientifically rigorous clinical trials is of utmost importance in further validating this treatment approach.

**Ethics and dissemination:**

We have submitted the clinical study protocol to the Ethics Committee, and it has been approved under ethical approval number 2021-1036-111-01. The results of the trial will be disseminated through peer-reviewed academic journals and presentations at professional conferences.

**Registration number:**

ChiCTR2100053556.

## Introduction

Anal fistula is a common, benign anorectal condition characterized by the formation of an abnormal tract connecting the rectum or anal canal to the perianal skin, often manifesting with symptoms such as perianal pain, purulent discharge, and pruritus [1, 2]. Approximately 90% of perianal fistulas are believed to originate from cryptoglandular sources, primarily resulting from infected anal glands. Other etiologies encompass inflammatory bowel disease (such as Crohn’s disease), tuberculosis, trauma, irradiation, or less common infections [3–5]. Digital rectal examination (DRE) is instrumental for the initial assessment of an anal fistula, providing insights into its location, size, consistency, and extent of infiltration. Complementing DRE, ultrasonography offers a dynamic, high-definition imaging solution for the evaluation of anal fistulas. Additionally, when assessing for potential malignancy or inflammatory bowel disease, colonoscopy is often indicated which could be conducted under sedation or general anesthesia to minimize patient discomfort and facilitate a thorough examination. However, magnetic resonance imaging (MRI) is considered the gold standard imaging modality for evaluating anorectal conditions, providing detailed images that enable the precise classification of anal fistulae based on their location and complexity [6]. Park’s classification, which is widely accepted in the field, delineates fistula tracts in relation to the anorectal musculature, categorizing them as intersphincteric, transsphincteric, suprasphincteric, and extrasphincteric [7]. This classification proves particularly valuable in identifying complex fistulas that originate from the high intersphincteric, high transsphincteric, extrasphincteric or suprasphincteric regions. In addition to that, the Garg classification introduced in 2017 stratifies anal fistulae into five grades according to their complexity and the extent of external sphincter involvement and also provide some guidances regarding the management of anal fistulae. Grades I and II are classified as simple, permitting safe fistulotomy without the risk of incontinence. In contrast, Grades III to V involve substantial sphincter engagement and are classified as complex, requiring sphincter-saving procedures to mitigate the risk of incontinence [8]. Among these cases, HHAF stands out as a notable example which represents a profound posterior anal fistula that extends into the ischiorectal fossa and forms a horseshoe-shaped tract with potential unilateral or bilateral extension [9, 10]. Given its unique anatomical features, the clinical management of HHAF proves challenging due to factors such as its deep location, intricate tract structure, and involvement of the sphincter [11, 12]. In Europe, the estimated incidence of anal fistula ranges from 1.2 to 2.8 per 10,000 individuals, with a peak occurrence observed between the ages of 20 and 40 years [13]. Epidemiological studies conducted in China indicate that anal fistula accounts for approximately 1.67% to 3.60% of anorectal disease cases, with a male-to-female ratio ranging from 5:1 to 6:1 [14]. According to statistical data, HHAF accounting for about 2%-5% of all anal fistulas [15, 16]. Despite its relatively rare occurrence, there exists a dearth of comprehensive literature addressing HHAF treatment options. However, it is widely acknowledged that surgical intervention serves as the cornerstone for achieving disease resolution.

Dr. Patrick H. Hanley developed the Hanley procedure grounded in the etiology, pathophysiology, and anatomical characteristics of complex horseshoe-shaped anal fistulas [17]. Subsequent scholars have further refined this procedure, such as preserving the external sphincter without excision and not tightening the suture after surgery. These modifications have established the modified Hanley procedure as a widely adopted surgical technique for treating HHAF [18, 19]. Nonetheless, challenges such as inadequate postoperative drainage and delayed wound healing persist. Other surgical alternatives such as fistulotomy, due to the potential risk of incontinence, are now less commonly employed for complex transphincteric fistulas [20]. Ligation of the intersphincteric fistula tract (LIFT) has become a s prevalent method for managing complex anal fistulas. Notably, the procedure demonstrates an impressive overall success rate of 76.5%, with only 1.4% of patients experiencing fecal continence impairment. It is important to note that the presence of horseshoe fistulas and a history of prior fistula surgeries emerged as risk factors associated with the diminished efficacy of LIFT [21]. With the advent of minimally invasive surgical techniques, various novel surgical and therapeutic strategies have emerged. These include the utilization of biological scaffold materials such as anal fistula plugs (AFP), which is simple, minimally invasive and with a short length of stay. However, the long-term healing rates of fistulas after AFP range from 38% to 56%, accompanied by fecal continence impairment in approximately 26.8% of patients [22, 23]. Additionally, video-assisted anal fistula treatment (VAAFT) provides long-term benefits by reducing anal incontinence while promoting earlier healing and accelerated rehabilitation, but it displays a wide variation in the healing rate of complex anal fistulas [24, 25]. Furthermore, fistula-tract laser closure (FiLaC) offers several advantages, including preserving the sphincter function and achieving a high primary healing rate, however, an excessively curved tract may contribute to an increased failure rate [26, 27]. At present, the incision and thread-drawing surgery, as a traditional method of anal fistula, is still the usual standard surgical treatment measure for HHAF [31]. It utilizes elastic tension to induce chronic sphincter ischemia, leading to gradual sphincter division. This method boasts a high surgical cure rate and results in few postoperative complications. However, the inward force from the ligature can occasionally cause severe pain in surrounding tissues, anal damage, and delayed healing [28]. The diversity of operations underscores the complexity of the disease and highlights the absence of a single optimal surgical technique for achieving ideal patient outcomes. Therefore, a suitable approach should combine various treatment methods.

Currently, our study focuses on validating the efficacy of a surgical procedure known as multi-incision and tube-dragging therapy combined with laser closure (MITD-LaC) for HHAF management. This technique is based on multi-incision and tube-dragging therapy (MITD), which involves the formation of only two incisions (semi-horseshoe anal fistula) or four incisions (complete horseshoe anal fistula) and the placement of multiple drainage tubes after the operation instead of conventional open incisions to preserve the anal sphincter as much as possible while ensuring sufficient wound drainage. This approach addresses the challenge of large postoperative wounds in HHAF surgery. Smaller incisions translate to minimal tissue damage and reduced scar formation post-surgery. Additionally, smaller incisions lower the likelihood of wound dehiscence during the postoperative healing process [29]. The interconnected drainage system between the incisions facilitates more efficient removal of necrotic and infectious materials, thereby promoting faster healing and tissue regeneration. It also includes subsequent postoperative measures, such as dragging, irrigation, and suction, to expedite the removal of necrotic tissue from the wound cavity [30]. Our previous study and related literature show that compared with incision and thread-drawing therapy (ITD), MITD has been demonstrated to reduce postoperative complications, shorten healing time, and effectively protect anal sphincter function [31, 32]. Furthermore, the utilization of laser closure in the MITD-LaC approach contributes to enhancing the efficacy. It represents a novel approach for preserving the sphincter function, as it combines the photothermal effect of laser energy to simultaneously disrupt the fistula epithelium and obliterate the remaining fistula track, resulting in closure of both internal and external orifices. The emitted laser energy induces tissue shrinkage and gradual sealing of fistula. The use of a suction catheter creates a dry environment for effective cutting through the fistula tract with the laser. Additionally, this vacuum effect created by suction generates negative pressure which promotes shrinkage of the tract and aids in achieving primary closure. By employing this technique, it effectively eliminates inflammatory epithelial and necrotic tissue on the fistula wall, presenting several advantages, such as reduced trauma, expedited recovery, and diminished postoperative pain [33–35].

The combination of MITD with LaC allows for the benefits of both procedures to be utilized concurrently. The MITD provides efficient drainage and sphincter preservation, while the LaC offers precise disruption of the fistula epithelium and obliteration of the tract, leading to effective closure. The theoretical basis for these benefits lies in the principles of wound healing and tissue management. Smaller incisions cause less disruption to the tissue architecture and preserve the blood supply, which is essential for the delivery of cells and nutrients necessary for healing. The laser therapy uses photothermal energy, which is known for its ability to induce immediate tissue shrinkage, thus facilitating the natural healing process by the body. In conclusion, the combined MITD-LaC approach for HHAF treatment theoretically seems to present several advantages over traditional methods, including improved sphincter preservation, enhanced wound healing, reduced complications, shortened healing time, and better pain management. To date, MITD-LaC has not been thoroughly evaluated in scientifically rigorous randomized controlled trials. Thus, it is critical to perform a randomized controlled trial to compare its efficacy with incision and thread-drawing therapy. Our primary objective is to rigorously evaluate the efficacy of the MITD-LaC for the treatment of HHAF. This includes assessing the wound healing time and comparing it against the ITD. Our secondary objectives include evaluating postoperative anal pain, fecal incontinence, incidence of postoperative complications, and quality of life in patients. These are designed to provide a comprehensive evaluation of the MITD-LaC technique and its impact on patient outcomes.

## Methods

### Study design and objectives

**Fig 1:**
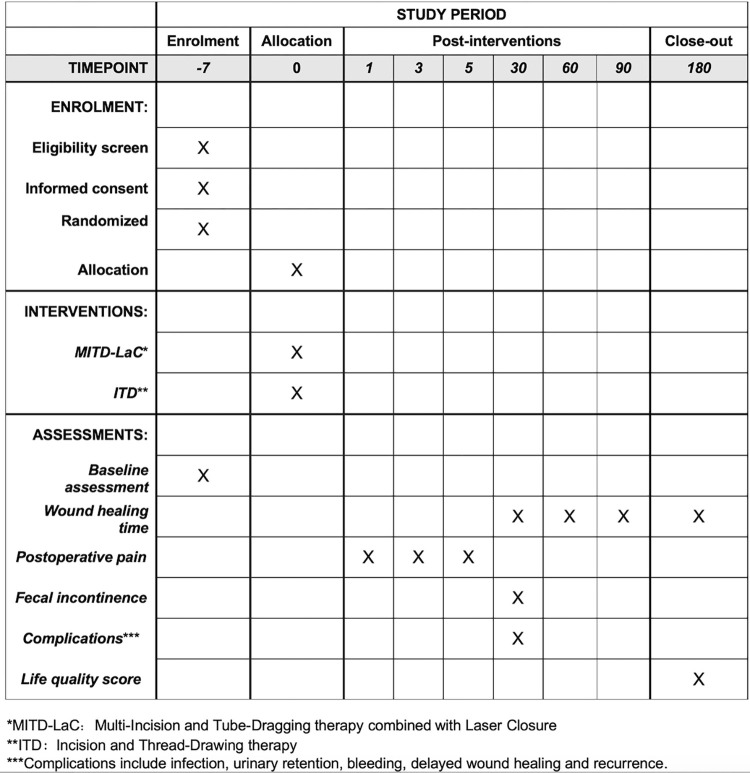
The schedule of enrolment, interventions, and assessments.

This study is designed as a prospective, randomized, controlled and interventional trial. The design aims to provide a rigorous framework for validating the efficacy and safety of MITD-LaC compared to traditional incision and thread-drawing therapy for the treatment of HHAF. Due to the unanticipated consequences of the COVID-19 pandemic and necessary adjustments within the research team, the recruitment period has been postponed. Consequently, it is now programmed to last around four months, beginning on June 30, 2024, and ending on November 1, 2024. The schedule of enrollment, intervention and assessment is displayed in Fig 1.

### Inclusion and exclusion criteria

Inclusion criteria are male or female hospitalized patients aged 18–65 years with HHAF, including complete horseshoe anal fistula and semi-horseshoe anal fistula which are diagnosed by perianal MRI imaging. Relevant diagnostic criteria will refer to the Clinical Practice Guideline for the Management of Anorectal Abscess, Fistulas in-Ano, and Rectovaginal Fistula (2016) by American Society of Colon & Rectal Surgeons (ASCRS) [36]. Patients must voluntarily agree to participate in the study, sign the informed consent form after undergoing an initial screening based on inclusion and exclusion criteria, and be willing and capable of completing the follow-up requirements on time. Concurrently, they must provide comprehensive previous clinical data to ascertain whether they meet the indications for anal fistula surgery and must also confirm that no surgical treatment has been performed and the disease duration is no more than half a year until now.

Exclusion Criteria:

- Patients aged below 18 or above 65 years.
- Pregnant or lactating women.
- Patients with malignant tumors detected by electronic colonoscopy, specific infections (such as tuberculosis, Crohn’s disease, HIV infection, etc.), or severe perianal diseases like mixed hemorrhoids, anal fissure, and perianal eczema.
- Patients with cognitive impairment or an inability to understand and provide informed consent.
- Patients concurrently enrolled in another clinical trial.

### Recruitment and randomization

**Fig 2:**
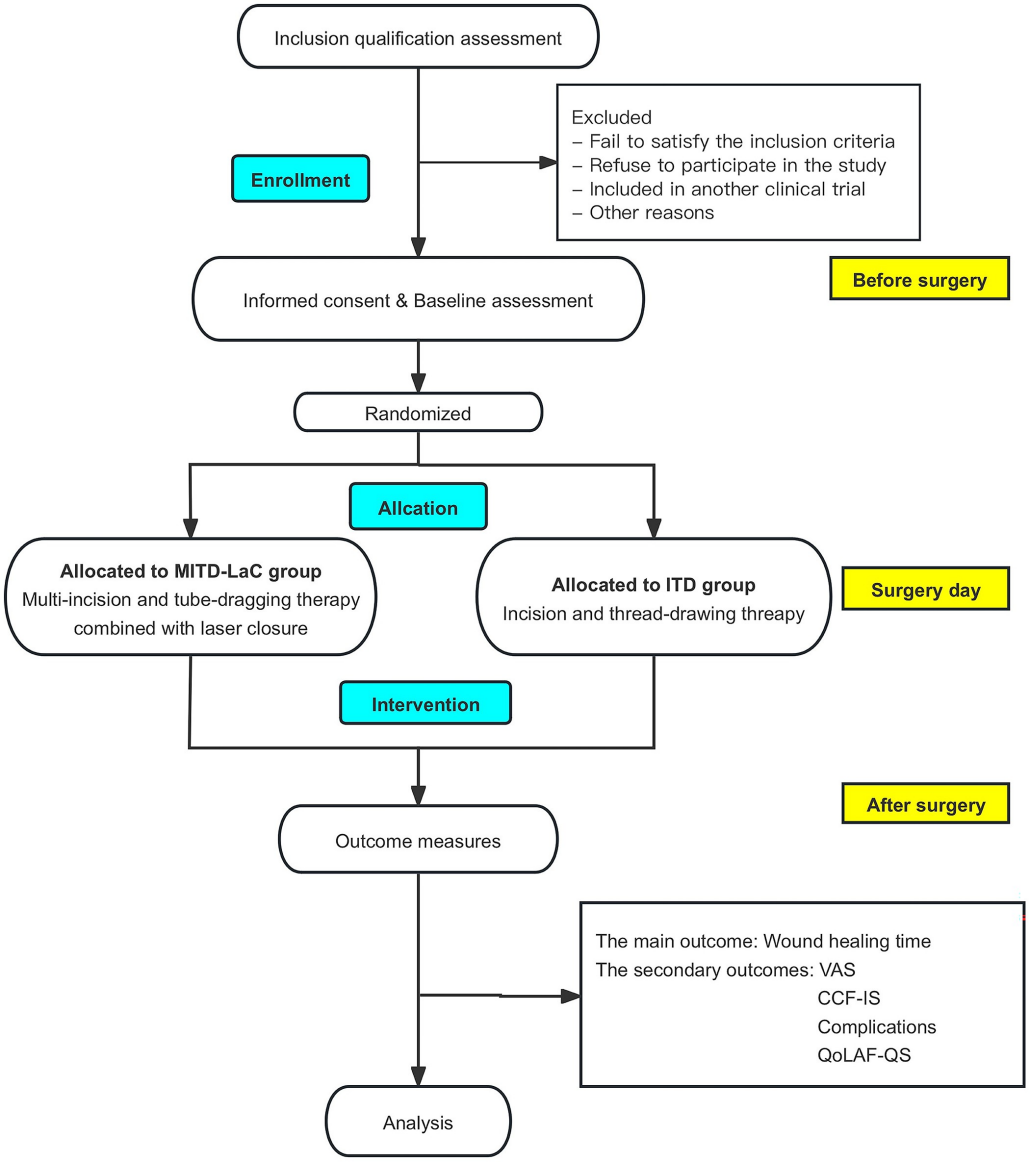
Trial flow chart.

Patients will be recruited, treated, and followed up at the Department of Anorectal, Shuguang Hospital Affiliated to Shanghai University of Traditional Chinese Medicine. All patients who express their willingness to participate in the trial will be provided with comprehensive information regarding the trial protocol and will be required to sign an informed consent form before proceeding. They will subsequently undergo randomization in a ratio of 1:1. The flow chart of study protocol is described in Fig 2.

Randomization will be performed using a random number table generated through SAS software by the trial’s assistant who will not participate in the subsequent research. Generate a table of random numbers for group allocation based on the number of cases. Allocate corresponding numbers to patients in the order of the time of signing the informed consent form, without arbitrarily altering the sequence of the numbers. Patients corresponding to each serial number will be randomly assigned to the different treatment groups based on the random number table. At the same time, this information needs to be kept properly to ensure transparency and traceability throughout the allocation process.

It is imperative to highlight that surgical procedures inherently involve significant physical interventions, such as incisions and post-operative dressing changes. The nature of these interventions complicates the implementation of blinding for trial personnel and challenges the ability to keep patients uninformed about the specific type of surgery they receive. Consequently, to ensure the fairness and objectivity of the research, we plan to establish a comprehensive Statistical Analysis Plan (SAP) before the start of the study. This plan is designed to maintain consistency and rigor in statistical analysis throughout the research process, thereby minimizing potential biases to the greatest extent possible.

### Interventions

#### Preoperative preparation

Newly enrolled patients will undergo a thorough evaluation that includes transrectal ultrasonography (TRUS) and an perianal enhanced magnetic resonance imaging (EMRI) to assess the extent of HHAF lesions and their relationship with surrounding tissues. The MRI and ultrasound images will be meticulously reviewed before surgery to identify the precise location of the internal opening, confirm the direction of the fistula, and establish its association with neighboring sphincter structures. Spinal anesthesia (SA), being the most commonly employed regional anesthetic technique for anorectal surgery, offers the advantages of prompt onset and offset. However, it also leads to prolonged hospitalization due to arterial hypotension as well as extensive sensory and motor block [37, 38]. Therefore, total intravenous anesthesia (TIVA) will be employed during surgery, as it offers advantages such as improved oxygenation and better exposure of the surgical field [39]. Additionally, utilizing the lateral decubitus position has been proven beneficial in reducing incidences of intraoperative respiratory depression and bucking [40]. Generally, standard intraoperative monitoring includes monitoring of vital signs (heart rate, blood pressure, oxygen saturation), electrocardiogram (ECG), and respiratory function. Given the use of total intravenous anesthesia (TIVA), close monitoring of the patient’s airway, breathing, and circulatory status would be essential.

#### MITD-LaC procedure

*Step 1—Initial Incision and fistula tract dissection*. A radial incision will be made along the trajectory of the fistula tract, starting from the external opening or the distal end of a blind fistula near the 10 o’clock lithotomy position. Subcutaneous tissues will be dissected using an ultrasonic scalpel to facilitate the separation of the fistula tract.

*Step 2—Radial incision for fistula access*. A radial incision will be executed 3cm from the anal margin at the 7 o’clock position. This incision will expose the previously dissected fistula tract, which will then be further separated towards the 6 o’clock position until near the lower margin of the levator ani muscle. An electrotome will be employed to open the fistula tract, and a silver probe will be inserted to assess the length of the remaining tract above the deep part of the external sphincter.

*Step 3—Laser ablation*. The laser device to be utilized is the LEONARDO® Mini 1470 nm, set to a 12W power and operated in "CW Mode (Continuous Wave Mode)." A fiber optic guide wire will be positioned at the top of the remaining fistula tract for ablation with a 1470nm wavelength laser. The ablation’s duration will need to be meticulously adjusted to optimize the delivery of laser energy, ensuring adequate heat penetration for the closure of the tract while minimizing overheating and protecting adjacent sensitive tissues. Multiple ablations may be conducted on thicker fistula ends to ensure thorough treatment. It will be critical to maintain an interval of more than 10 seconds between each ablation session.

*Step 4—Fistula tract excision and debridement*. The fistula tract will be excised, followed by the removal of inflammatory tissues near the internal opening using a curette.

*Step 5—Management of the contralateral fistula tract*. The same methodology will be applied to the contralateral fistula tract. A radial incision will be made at the distal end of the fistula tract near the 2 o’clock position, with subcutaneous tissues being dissected to expose and separate the tract. A radial incision at the 5 o’clock position will facilitate further dissection of the tract up to the 6 o’clock position before excision. This will culminate in the creation of four incisions, one extending to the deep part of the external sphincter.

*Step 6—Separation of fibrous scar tissues*. Blunt dissection will be utilized to separate fibrous scar tissue interstices between incisions, accompanied by the removal of inflammatory and necrotic tissues, ensuring unobstructed inter-incisional connectivity.

*Step 7—Wound management and drainage installation*. After the excision of the fistula tract and inflammatory tissue debridement, comprehensive hemostasis will be achieved. All surgical sites will be irrigated with a 3% hydrogen peroxide solution for disinfection. Subsequently, 16F/18F rubber drainage tubes will be placed between the interconnected incisions and loosely secured to facilitate wound drainage and healing. The internal anal wound will be packed with gauze, while external wounds will be dressed with antiseptic cotton pads under compression.

**Fig 3:**
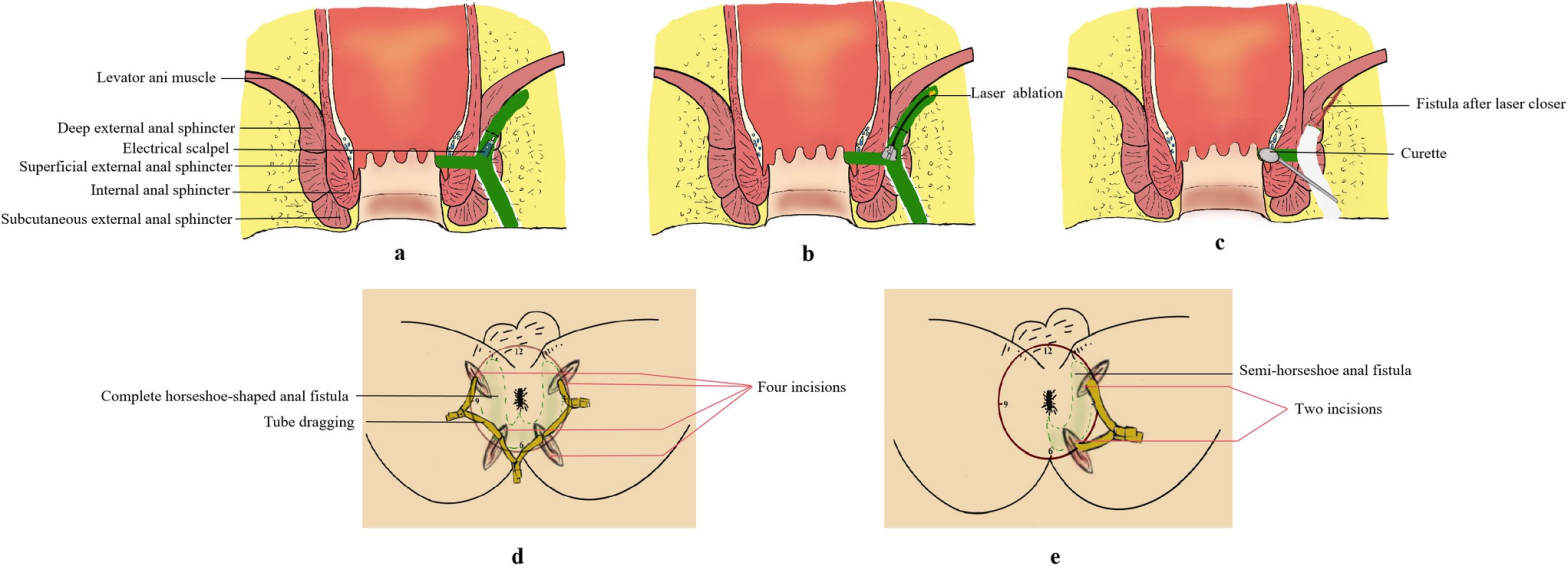
The operation diagram of MITD-LaC therapy. (a) Incise and dissect the fistula to the lower edge of the levator ani muscle. (b) Laser ablation of remaining deep fistula tract. (c) Curettage near the internal opening. (d) Four incisions and drainage tubes installation after MITD-LaC in complete horseshoe-shaped anal fistula. (e) Two incisions and drainage tubes installation after MITD-LaC in semi-horseshoe anal fistula. MITD-LaC = multi-incision and tube-dragging therapy combined with laser closure.

**Fig 4:**
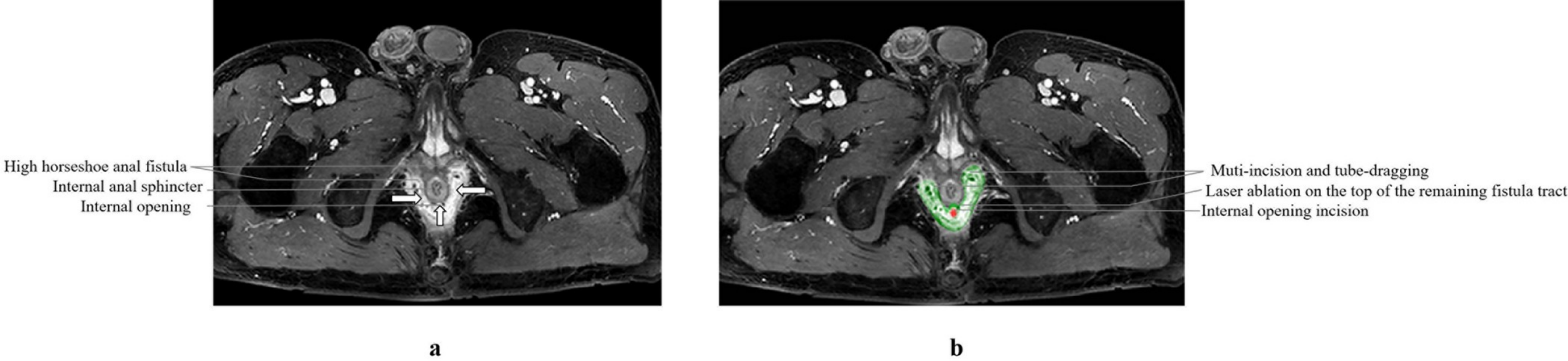
The schematic operation diagram of MRI. (a) The diagram of preoperative MRI. (b) Muti-incision and tube-dragging on the HHAF tracts, the internal opening incision and the laser ablation adopted on the top of the remaining fistula tract. HHAF = high horseshoe-shaped anal fistula.

For semi-horseshoe anal fistulas, the procedure will incorporate Steps 1 to 4, and 6 to 7, resulting in two incisions, one of which penetrates deeply to the external sphincter. The MITD-LaC procedure is displayed in Figs 3 and 4.

#### ITD procedure

*Step 1 –Incision of fistula tract*. An incision will be made to excise the low-lying fistula tracts and their branches from the external openings or the distal end, cutting through the subcutaneous and superficial parts of the external sphincter, and the lower edge of the internal sphincter near the internal opening. This will involve clearing all residual cavities and necrotic tissues.

*Step 2—Creation of artificial external opening*. A radial incision will be made on the perianal skin near the internal opening, around the 6 o’clock in lithotomy position. This step exposes the fistula, forming an "artificial external opening." A curette will then be used for thorough debridement around the internal opening.

*Step 3—Thread-ligating formation*. A ball-tipped probe will be inserted into the fistula via the artificial external opening, guided by a finger inside the anus to emerge at the internal opening. A rubber band will be attached to the probe’s tail end and pulled out through the anus, allowing the rubber band to pass through the fistula and be tightened, creating a thread-ligating.

*Step 4—Wound packing and dressing*. After irrigating and achieving hemostasis, the anal canal will be packed with Vaseline gauze. The incision will then be covered with gauze and bandaged.

**Fig 5:**
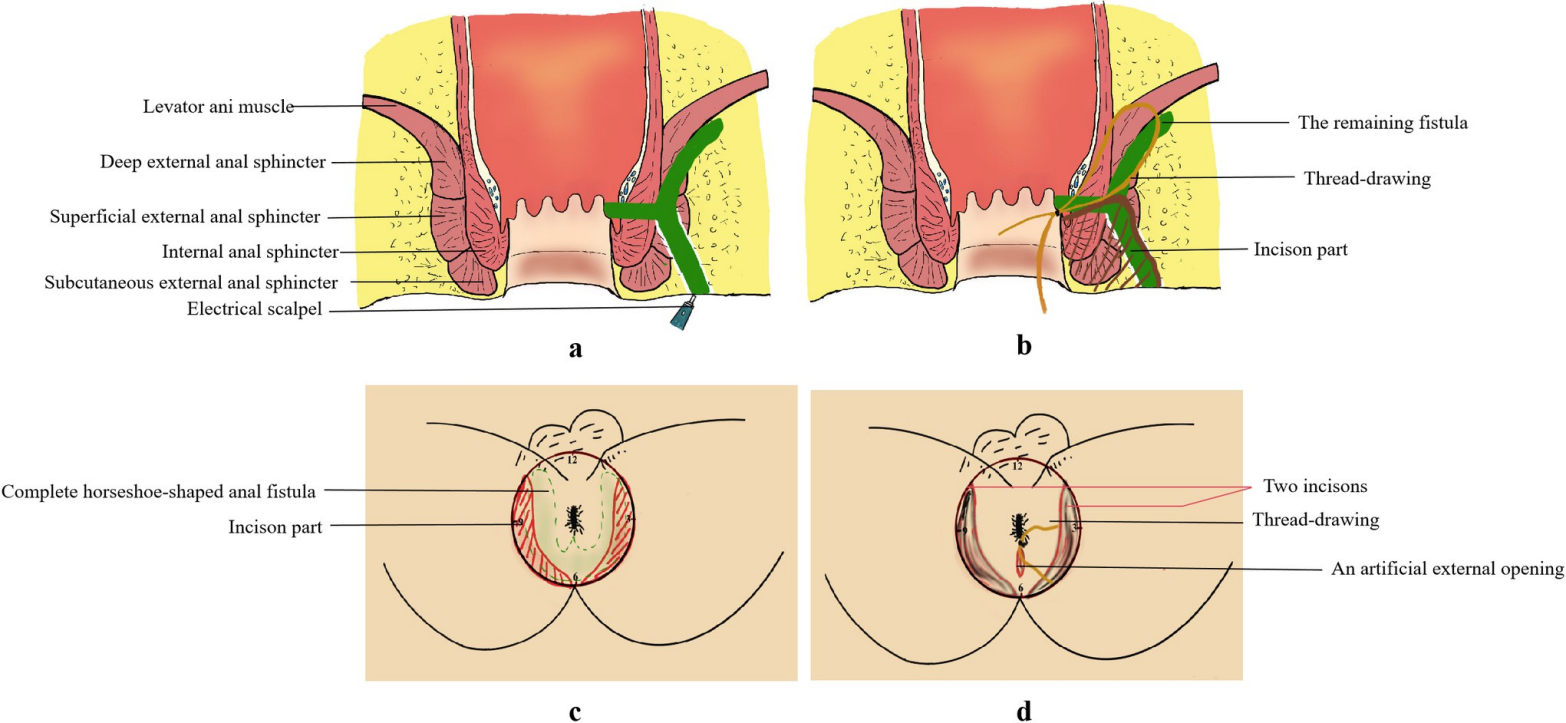
The operation diagram of ITD therapy. (a) Incise the fistula tracts from the external openings or the distal end. (b) Thread-drawing formation. (c) Extent of incisions in the lithotomy position. (d) Incisions and thread-drawing after ITD. ITD = incision and thread-drawing.

The above describes the ITD procedures of the complete horseshoe-shaped anal fistula. For semi-horseshoe anal fistula, only one side of the fistula will be incised in the first step. The operation diagram of ITD is showed in Fig 5.

#### Postoperative wound management

Both groups will initiate dressing changes on the first postoperative day, accompanied by routine cleansing of the anal canal and wound cavity. In the MITD-LaC group, to ensure optimal postoperative drainage, the appropriate timing for the removal of drainage tubes is important. Typically, based on the progression of granulation tissue growth, the gradual removal of drainage tubes is initiated around one week post-operation, starting from the distal sections and proceeding in stages towards the proximal end near the internal opening by approximately the second postoperative week. After tube removal, the application of cotton pad compression is recommended to encourage the adhesion of granulation tissues within the surgical cavity, thereby facilitating wound healing. Additionally, vigilant monitoring for pseudohealing is necessary, which is characterized by the healing of the external wound layer while the internal layer remains unhealed. In cases where the deep wound layer fails to heal repeatedly, leading to cavity formation, expanding the wound and enhanced drainage may be required to ensure complete healing. In the control group, the threads will typically fall off around two weeks after surgery. Gentle pulling is recommended to prevent them from adhering to the wound granulation. If they do not naturally fall off after more than two weeks or if there is loosening of the initially tightened threads, a secondary tightening procedure should be performed.

The patients will receive standard postoperative care in the hospital. If there are no bleeding, infection, or other severe adverse events, the patients will be discharged about one week after surgery. Simultaneously, they will be instructed to attend regular follow-up appointments at the outpatient clinic to monitor wound healing at intervals of 1, 2, 3, and 6 months post-surgery.

### Study outcomes

The primary outcome measure is wound healing time, defined as the duration from the day of surgery to complete epithelialization of the surgical wound [41]. Wound healing will be confirmed through local physical examination and a comprehensive assessment by the physician during outpatient follow-up visits.

Secondary outcomes include the assessment of postoperative pain levels on days 1, 3, and 5 using a visual analogue scale (VAS). Additionally, Cleveland Clinic Florida Incontinence Score (CCF-IS) will be employed to assess fecal incontinence scores within 30 days post-surgery. Postoperative complications, such as infection, urinary retention, bleeding, delayed wound healing, and recurrence within one month after surgery, will also be observed. Furthermore, the Quality of Life in Patients with Anal Fistula Questionnaire Score (QoLAF-QS) will be utilized to evaluate patients’ quality of life six months following the operation.

Postoperative infection is characterized by significant pain, swelling, and pustulethe formation at the surgical site, which may be accompanied by fever. This condition necessitates the initiation of antibiotic therapy [42]. Postoperative urinary retention is defined as the inability to void spontaneously in the presence of bladder overdistension, necessitating catheterization for relief [43]. Postoperative hemorrhage is marked by excessive bleeding that demands immediate intervention, such as localized pressure for hemostasis at the site of bleeding [44]. Delayed wound healing is identified as a failure of the wound to achieve complete re-epithelialization within 3 months post-surgery. Recurrence of a fistula is identified when it re-emerges around the prior surgical site, often necessitating the use of Transrectal Ultrasound (TRUS) or Endorectal Magnetic Resonance Imaging (EMRI) for diagnostic confirmation [45, 46].

### Statistical considerations

#### Estimation sample size

The sample size for this study is calculated using a two-sample comparison of means. Previous research has reported that the average healing time for patients treated with incision and thread-drawing therapy for HHAF is 62.69±3.54 days [47]. In our study, we assume a reduction in average healing time by 3 days in the MITD-LaC group, with an allowable error (δ) of 3 days and a known standard deviation of 3.54 days. The study is powered to an α = 0.05 (type I error rate) and β = 0.10 (type II error rate), leading to Z_α_ = 1.960 and Z_β_ = 1.282, as determined from statistical tables.

Based on the calculation, at least 25 participants are required for each group, as computed using Power Analysis and Sample Size Program 2021 (PASS, NCSS Statistical Software, Kaysville, UT, USA). To account for an anticipated dropout rate of 20%, we aim to recruit a total of 64 participants for both groups, thus allocating 32 participants per group. This sample size will ensure adequate power to detect clinically meaningful differences in healing time between the two treatment approaches.

#### Statistical analysis

SPSS software (IBM SPSS 17.0, SPSS Inc) will be used for statistical analysis of the collected data. The normality of measurement data is verified by Shapiro-Wilk test, and the homogeneity of variance is assessed using Levene’s test. All statistical analyses will employ two-sided tests, with P-values less than 0.05 considered statistically significant.

Baseline data will be collected for both patient groups, encompassing gender, age, disease duration, occupation, body mass index (BMI), smoking status, alcohol consumption, prior medications, and history of previous perianal surgery, to ensure comprehensive demographic and clinical profiling. Among these variables, gender, occupation, smoking status, alcohol consumption, prior medications, and history of previous perianal surgery are categorical, and will be presented as patient counts and respective percentages, with intergroup comparisons performed using the Chi-square test. Continuous variables such as age, disease duration, and BMI, if normally distributed and with homogeneity of variance, will be expressed as mean ± standard deviation and compared between groups using the independent samples t-test. Otherwise, they will be presented as medians, with the Mann-Whitney U test employed for statistical analysis.

The primary outcome, wound healing time, along with secondary outcome scores for VAS, CCF-IS, and QoLAF-QS, are continuous data. If they follow a normal distribution with equal variances, they will be described using the mean and standard deviation. Otherwise, they will be described using the median and interquartile range. Comparisons between groups for QoLAF-QS will be conducted using the independent samples t-test or Mann-Whitney U test, depending on the normality and homogeneity of the data. The incidence of complications, being categorical data, will be analyzed using the Chi-square test. Efficacy indicators that require repeated measurements, such as VAS and CCF-IS scores, will be analyzed using repeated measures ANOVA and a repeated-measures mixed effects model. In this model, the surgical method will be included as a fixed effect to assess its universal impact across all patients. Simultaneously, individual differences such as age, sex, and disease history will be incorporated as random effects to examine how these personal factors influence each patient’s treatment response. This analysis aims to provide insights into the varied effects of the MITD-LaC treatment across different patient populations and inform the optimization of treatment strategies based on individual variances. All data and analyses will be reported follow CONSORT guidelines.

#### Data collection and quality management

We will employ an electronic data capture (EDC) system for efficient data management, with a dedicated clinical research assistant responsible for overseeing both data management and study progress monitoring. The research assistant will access the EDC system through a strictly confidential personal electronic account, thereby ensuring the secure input and transmission of collected patient information to the central database. To safeguard patient confidentiality, all participant study data will be anonymized. This will be overseen by the same research assistant, who will be responsible for all aspects of data entry, thorough checking, and meticulous data management.

### Ethics and dissemination

#### Approval

The clinical study protocol was submitted to the IRB of Shuguang Hospital affiliated with Shanghai University of TCM for approval which implements an annual follow-up review system for the ethical approval of clinical studies. Each ethical approval is valid for a duration of one year. We have obtained the written ethical approval and full supervision on November 02, 2021. Ethics Committee Approval number: 2021-1036-111-01. The latest ethical approval update is valid from November 2, 2023, to November 01, 2024, with the Approval Number: 2021-1036-111-03. We will continue to apply for extensions of the ethical approval to ensure ongoing compliance. This project has been registered and submitted to Shanghai Science and Technology Commission with the registration number of ChiCTR2100053556. Any substantial changes or modifications to the informed consent form will be subject to review by an independent medical ethics committee. Considering the clinical nature of the study and the potential risks associated with the intervention, an independent Data and Safety Monitoring Committee (DSMC) will conduct periodic reviews of the study’s progress and oversight. Strict adherence to medical ethical principles will be enforced to safeguard patient privacy and ensure the safety of participants.

#### Patient informed consent

Before participating in the study, written informed consent will be diligently obtained from all patients. Patients will receive comprehensive information regarding the study both orally and in written form, ensuring that they fully understand the relevant details. Their voluntary participation in the study will be unequivocally emphasized.

Patients will also be explicitly informed about the privacy requirements and will provide consent for direct access to their previous medical data. Moreover, patients will be made aware of their right to withdraw from the trial at any time, and no reason will be required for such a decision.

#### Dissemination

The results of the study will be disseminated in a peer-reviewed journal. Additionally, the results will be presented at suitable national and international conferences. Pertinent information regarding the trial and its outcomes will be shared with patients and disseminated through social media.

## Discussion

Currently, there remains a lack of consensus regarding the surgical management of HHAF. While complete resection of the fistula is not the sole objective or optimal choice of surgery, achieving cure can be accomplished by ensuring thorough removal of primary infection and internal opening, along with maintaining unobstructed wound drainage [48, 49].

The MITD-LaC technique is designed to optimize the preservation of normal tissue and sphincter function. Therefore, we believe that this new method can better promote wound healing, with lower incidence of postoperative complications and higher patient satisfaction. Unlike conventional approaches that involve one or two large incisions, it employs multiple smaller incisions to minimize the wound area and subsequent scar formation. These incisions are interconnected by a biocompatible drainage system, effectively reducing the risk of foreign body reactions and inflammation in the surrounding tissues. Furthermore, the smooth surface of the drainage system facilitates easy maneuverability between the incisions, promoting efficient cleaning of subcutaneous wound cavities and also further lowering the risk of infection. Our previous study has confirmed that MITD can significantly reduce postoperative complications, shorten healing time, and protect the function of anal sphincter [31]. In addition, laser technology is utilized for the cauterization of fistula tracts. Exposure to laser energy results in denaturation and necrosis of epithelial cells within the fistula tract. Inflammatory and necrotic tissues are subsequently dissolved and absorbed, while any remaining debris is drained through an external opening. The resulting void is filled with fresh granulation tissue before the closure of the fistula. This minimally invasive technique leads to reduced intraoperative trauma, less bleeding, and accelerated postoperative wound healing [27].

This study has several strengths. It is the first one to combine multi-incision and tube-dragging therapy (MITD) with laser closure (LaC), and also the first randomized controlled trial to evaluate its safety and efficacy. As such, it will contribute high-quality, peer-reviewed evidence to guide clinical practice, filling a critical gap in the existing medical literature. Additionally, the trial includes comprehensive outcome measures that assess both subjective and objective aspects. By adopting such a multifaceted approach to outcome evaluation, the study ensures a more holistic understanding of the efficacy of MITD-LaC therapy on patients.

However, there are some limitations to the study. As a pilot trial with a limited sample size designed to gather preliminary data, the study’s capacity for controlling potential confounding factors is somewhat restricted. Although we incorporate demographic and clinical characteristics such as age, gender, and disease duration into our baseline analysis, the scope of our study does not extend to a comprehensive examination of all possible confounders. Mei Z et al. conducted an international, evidence-based Delphi consultation survey to identify potential risk factors for recurrence following anal fistula surgery. These risk factors are divided into three main categories: patient-related risk factors, which encompass inflammatory bowel disease and the use of immunosuppressive medications; fistula-related factors, including characteristics like trans-sphincteric fistulas, the total number of fistulas, and the height of the internal opening; and factors related to the surgery itself, such as the surgical approach employed and history of previous fistula surgeries [50]. Factors such as comorbid conditions, genetic predispositions, and he height of the internal opening, which could influence surgical outcomes, are not explicitly analyzed in our study protocol. The absence of this in-depth analysis may limit the objective appraisal of the MITD-LaC technique’s efficacy. However, our study’s randomized controlled trial design, by randomly assigning participants to either the intervention or control group, ensures an equal distribution of both known and unknown confounding variables across the groups. This randomization process is crucial for maintaining the internal validity of the study, enabling us to attribute differences in outcomes more confidently to the intervention itself rather than to external variables. Additionally, the postoperative follow-up period is confined to a duration of six months, lacking in extended long-term observational data. And the study’s design, being single center, also hampers the broad application and generalizability of the results. Thus, further verification is necessary through multi-center and large-sample clinical trials. It is also pertinent to mention that given the substantial variances in interventions, blinding of both investigators and participants may prove to be impracticable. Such a constraint might pave the way for assessment bias, particularly with subjective outcomes like the VAS scores for pain. Nevertheless, to mitigate the impact of any potential biases, it is imperative that investigators take proactive steps in thoroughly educating all participants across groups regarding the study protocol, operational procedures, and the proper implementation of the VAS for pain evaluation. At the same time, in assessing post-operative anal function, we will utilize only the traditional Cleveland Clinic Florida Incontinence Score, but we also noticed some new scoring systems to assess fecal incontinence, for example the Garg Incontinence Scores (GIS) [51]. GIS enhances the robustness of the scoring while maintaining simplicity in its application, shifting from a surgeon-centric to a patient-oriented evaluation, which represents a logical and significant advancement. Moreover, GIS addresses previously neglected incontinence types, including stress, mucus, and urge fecal incontinence, thus providing a more comprehensive and inclusive assessment [52]. The reason why we have decided not to incorporate the GIS scoring system as a secondary outcome in our study is based on the GIS scoring system being a relatively new assessment tool that has not yet been sufficiently validated in our specific research context. Additionally, adding the GIS scoring system would require further ethical approvals and data analysis, potentially imposing additional pressures on our research progress and resources. However, we recognize the potential value of the GIS scoring system. We believe it could have significant applications in future large-scale, multicenter randomized controlled trials. We will continue to monitor research developments in the field of GIS scoring systems, staying updated with the latest findings and applications. Once conditions are favorable, we will consider applying the GIS scoring system to enhance our trial assessments in future large-scale, multicenter randomized controlled trials.

MITD-LaC has a broad application prospect in the treatment of HHAF, especially suitable for patients who are concerned about postoperative complications such as fecal incontinence and those seeking minimally invasive treatment options. But its safety and efficacy still need to be further validated in large sample, multi-center randomized controlled clinical trials. Moreover, long-term recurrence rate observation and cost-effectiveness evaluation should be enhanced for comprehensive assessment. Therefore, it is imperative to acquire more definitive and authoritative evidence via scientifically rigorous clinical trials to guide its clinical application.

## Supporting information

S1 File SPIRIT checklist. (DOCX)

S2 File Study protocol approved by the ethics committee (in English). (PDF)

S3 File Study protocol approved by the ethics committee (in Chinese). (PDF)

## Abbreviations

HHAF: high horseshoe-shaped anal fistula
MITD-LaC: multi-incision and tube-dragging therapy combined with laser closure
ITD: incision and thread-drawing therapy
VAS: visual analogue scale
CCF-IS: Cleveland Clinic Florida Incontinence Score
QoLAF-QS: Quality of Life in Patients with Anal Fistula Questionnaire Score
TRUS: transrectal ultrasonography
EMRI: enhanced magnetic resonance imaging
TIVA: total intravenous anesthesia
EDC: electronic data capture system
SAS: statistical analysis system
ASCRS: American Society of Colon & Rectal Surgeons
VAAFT: video-assisted anal fistula treatment
LaC: laser closure
MRI: magnetic resonance imaging
PASS: Power Analysis and Sample Size Program
SPSS: Statistical Package for the Social Sciences
DRE: digital rectal examination
SAP: statistical analysis plan
DSMC: data and safety monitoring committee
GIS: Garg Incontinence Scores
