# Supplementary material for: Evaluating the efficacy of multi-incision and tube-dragging therapy combined with laser closure for high horseshoe-shaped anal fistula: Protocol of a prospective, randomized, controlled trial
Source: PLoS One. 2024 Sep 27;19(9):e0307653. doi: 10.1371/journal.pone.0307653 (PMC11432866; doi:10.1371/journal.pone.0307653)
Supplement: S2 File — (PDF) [file pone.0307653.s002.pdf]

# Evaluating the Efficacy of Multi-incision and Tube-dragging Therapy Combined with Laser Closure for High Horseshoe-Shaped Anal Fistula: Protocol of a Prospective, Randomized, Controlled Trial

**Principle Investigator:** Prof. De Zheng, professor of the Department of Anorectal Surgery, Shuguang Hospital, Shanghai University of Traditional Chinese Medicine.

**E-mail:** zd1232@sina.com

**Address:** 528 Zhangheng Road, Shanghai 201203, China

**Version:** 1.0

**Date:** November 2, 2021

# Abstract

**Introduction：** High horseshoe-shaped anal fistula (HHAF) is a complicated and challenging condition that presents considerable obstacles in treatment. We are presently investigating a novel surgical technique involving a combination of multi-incision and tube-dragging therapy, and laser closure (MITD-LaC) for the management of HHAF. Due to the current scarcity of rigorous evidence evaluating this approach, it is essential to perform a well-designed randomized controlled trial to compare the effectiveness of this new method with incision and thread-drawing therapy.

**Methods and analysis：** This trial is a prospective, randomized, controlled and interventional study. After preliminary screening of qualified outpatients, a total of 64 adult patients will be enrolled in the trial and randomly allocated to either the MITD-LaC group or the control group (n=32 per group). These patients will receive either MITD-LaC or incision and thread-drawing therapy. The design aims to allow for a robust comparison between the two treatment modalities. The primary endpoint is the wound healing time, while secondary endpoints include postoperative anal pain at 1，3，and 5 days（measured with visual analogue scale)，fecal incontinence score within 30 days after operation (measured with Cleveland Clinic Florida incontinence score), and the occurrence of postoperative complications within 1 month after surgery, and quality of life up to six months postoperatively (evaluated by The Quality of Life in patients with Anal Fistula Questionnaire Score).

**Discussion：** This study represents the first randomized controlled trial evaluating the short-term outcomes of MITD-LaC, thereby aiming to contribute high-quality evidence to guide clinical

practice. Moreover, this trial incorporates comprehensive outcome measures assessing both subjective and objective dimensions. Because of this multidimensional assessment, MITD-LaC offers a promising potential for broader application in the treatment of HHAF. Consequently, obtaining more definitive and authoritative evidence through scientifically rigorous clinical trials is of utmost importance in further validating this treatment approach.

# Study Protocol

## 1.Study background

Anal fistula is a prevalent, non-malignant anorectal condition characterized by the development of an abnormal channel linking the rectum or anal canal to the perianal skin, often presenting symptoms like perianal discomfort, purulent discharge, and itching. High horseshoe-shaped analfistula (HHAF) is a type of deep posterior analfistula that extends into the ischiorectal space and creates a horseshoe-shaped passageway on one or both sides[1, 2]. Due to its distinctive anatomical features, managing HHAF clinically is a formidable task, primarily because of its profound location, intricate tract structure, and involvement of the sphincter muscles[3-5].

Our current research is centered on validating the effectiveness of a surgical procedure known as Multi-Incision and Tube-Dragging Therapy combined with Laser Closure (MITD-LaC) for managing High Horseshoe-Shaped Anal Fistula (HHAF). This technique is rooted in Multi-Incision and Tube-Dragging Therapy (MITD), which involves placing multiple drainage tubes instead of traditional open incisions to maximize the preservation of the anal sphincter while ensuring adequate wound drainage. The procedure also incorporates postoperative measures, such as dragging, irrigation, and suction, to expedite the removal of necrotic tissue from the wound cavity. Compared to Incision and Thread-Drawing Therapy (ITD), MITD has demonstrated the ability to reduce postoperative complications, shorten healing time, and effectively safeguard anal sphincter function[6, 7]. Additionally, the integration of laser closure in the MITD-LaC approach contributes to enhancing its effectiveness. This technique efficiently eliminates inflammatory epithelial and necrotic tissue on the fistula wall, offering several advantages, including reduced

trauma, accelerated recovery, and reduced postoperative pain.

Up to date, MITD-LaC has not undergone a comprehensive evaluation through scientifically rigorous randomized controlled trials. Therefore, it is imperative to conduct a randomized controlled trial to compare its efficacy with that of Incision and Thread-Drawing Therapy.

**2.Study object**

Before the enrollment, each patient will undergo magnetic resonance imaging (MRI) and transrectal ultrasound (TRUS) examinations to determine the extent of HHAF lesions and their relationship with surrounding tissues. The diagnosis of HHAF will be made and confirmed by at least two advanced radiology experts. Inclusion criteria will include male or female inpatients aged 18 to 65 years with HHAF, including complete horseshoe anal fistula and semi-horseshoe analfistula. Relevant diagnostic criteria will be referenced from the Clinical Practice Guideline for the Management of Anorectal Abscess, Fistulas in-Ano, and Rectovaginal Fistula (2016) by the American Society of Colon & Rectal Surgeons (ASCRS)[8]. Exclusion criteria will encompass patients below 18 or above 65 years of age, pregnant or lactating women, patients with malignant tumors detected through electronic colonoscopy, specific infections (such as tuberculosis, Crohn's disease, HIV infection, etc.), or severe perianal diseases like mixed hemorrhoids, anal fissure, and perianal eczema, patients with cognitive impairment or those unable to comprehend and provide informed consent, as well as patients concurrently enrolled in another clinical trial.

**3.Study design**

This study is designed as a prospective, randomized, controlled and interventional trial. 64 patients with HHAF will be included and randomly divided into MITD-LaC group and ITD group according to different surgical treatment methods, during which clinical data related to patients will be collected and prospective analysis will be performed.

**4.Sample size estimation**

The sample size for this study is calculated using a two-sample comparison of means. Previous research has reported that the average healing time for patients treated with incision and thread-drawing therapy for HHAF is 62.69 ± 3.54 days[9]. In our study, we assume a reduction in average healing time by 3 days in the MITD-LaC group, with an allowable error (δ) of 3 days and a known standard deviation of 3.54 days. The study is powered to an $\alpha=0.05$ (type I error rate) and $\beta=0.10$ (type II error rate), leading to $Z_{\alpha}=1.960$ and $Z_{\beta}=1.282$, as determined from statistical tables. Based on the calculation, at least 25 participants are required for each group, as computed using Power Analysis and Sample Size Program 2021 (PASS, NCSS Statistical Software, Kaysville, UT, USA). To account for an anticipated dropout rate of 20%, we aim to recruit a total of 64 participants for both groups, thus allocating 32 participants per group.

Randomization will be performed using a random number table generated through SAS software, which will be based on the total number of cases. The treatment allocation will correspond to serial numbers Q01-64, creating an overall random code table. These numbers will be assigned in accordance with the order of outpatient visits.

**5.Interventions**

(1) MITD-LaC procedure

A 1.5 cm incision will be made at the distal end of the perineal anal fistula, and the subcutaneous tissue will be separated to expose the fistula. Following the path of the fistula, the fistula tract will be carefully dissected to the posterior anal margin, at which point a radial incision will be made. For complete HHAF, the same procedure will be performed, extending the dissection towards the contralateral anal margin until reaching the internal opening. Subsequently, the fistula will be excised, and all surrounding inflammatory and necrotic tissue will be

meticulously removed.The fistula located above the dental line will be incised, and an optical fiber guide wire will be placed into the upper portion of the fistula tract, followed by laser vaporization and ablation of the fistula tract.After achieving hemostasis and performing thorough wound irrigation, a rubber drainage tube will be carefully inserted between the adjacent incisions and firmly secured in position. The wall of the rubber drainage tube should be perforated with multiple small holes, each approximately 2mm in diameter, thereby optimizing drainage efficiency.

(2) ITD procedure

The fistula tracts and abscess cavities located below the dentate line will be excised or excoriated, the wound margin will be debrided, and it will be ensured that any inflammatory and necrotic tissues within the tracts will be extirpated. At the same time, the fibrotic wall and scar tissue around the external opening should be removed completely.A radial incision will be made about 3cm on the anal margin at the internal opening position, followed by incision and curettage of the fistula tract to remove any necrotic tissue and completely excise both the internal opening and associated infectious lesions.After open drainage of the wound, a probe will be inserted into the internal opening and guided out through the anus. A rubber band will be then attached to the end of the probe and pulled out of the anus. Subsequently, the two ends of the rubber band should be pulled tightly and then fixed with silk thread.The wound margin will be trimmed, and then compression hemostasis and packing will routinely be performed. Subsequently, standard bandaging and fixation procedures will be carried out to secure the wound area.

**6.Clinical observation index**

(1) Baseline data statistics

Baseline information (such as gender, age, height, weight, primary/recurrent, etc.) will be

recorded for both groups before surgery.

(2) Primary outcome measure

The primary outcome measure is wound healing time, defined as the duration from the day of surgery to complete epithelialization of the surgical wound[10].Wound healing will be confirmed by the doctor's local examination and integrative evaluation during the patient's regular outpatient follow-up after surgery.

(3) Secondary outcome measure

Secondary outcomes include the assessment of postoperative pain levels on days 1, 3, and 5 using a visual analogue scale (VAS). Additionally, the Cleveland Clinic Florida Incontinence Score (CCF-IS) will be employed to assess fecal incontinence scores within 30 days post-surgery. Postoperative complications, such as infection, urinary retention, bleeding, delayed wound healing, and recurrence within one month after surgery, will also be observed. Furthermore, the Quality of Life in Patients with Anal Fistula Questionnaire Score (QoLAF-QS) will be utilized to evaluate patients' quality of life six months following the operation.

The occurrence of postoperative infection is defined as the presence of obvious pain accompanied by swelling and pustulation at the surgical site, with or without fever, and necessitating administration of antibiotic therapy. Postoperative urinary retention is defined as the inability to void spontaneously in the presence of bladder overdistension, necessitating catheterization for relief[11]. Postoperative bleeding is defined as excessive bleeding that requires urgent management like local pressing hemostasis in the bleeding site[12]. Delayed wound healing is defined as any wound that has not achieved complete re-epithelialisation by 3 months[13]. Recurrence is defined as a fistula that reoccurs around the previous surgical site, and

often requires the utilization of TRUS or EMRI for diagnostic assistance[14, 15].

**References**


1. Pezim ME. Successful treatment of horseshoe fistula requires deroofing of deep postanal space. Am J Surg. 1994;167(5):513-5. doi: 10.1016/0002-9610(94)90247-x. PubMed PMID: 8185039.

2. Usui A, Ishiyama G, Nishio A, Kawamura M, Kono Y, Ishiyama Y. Two-Stage Complete Deroofing Fistulotomy Approach for Horseshoe Fistula: Successful Surgery Leaving Continence Intact. Ann Coloproctol. 2021;37(3):153-8. Epub 20210112. doi: 10.3393/ac.2020.06.08. PubMed PMID: 33445836; PubMed Central PMCID: PMCPMC8273714.

3. Browder LK, Sweet S, Kaiser AM. Modified Hanley procedure for management of complex horseshoe fistulae. Tech Coloproctol. 2009;13(4):301-6. Epub 20091008. doi: 10.1007/s10151-009-0539-6. PubMed PMID: 19813077.

4. Jacob TJ, Perakath B, Keighley MR. Surgical intervention for anorectal fistula. Cochrane Database Syst Rev. 2010;(5):CD006319. Epub 20100512. doi: 10.1002/14651858.CD006319.pub2. PubMed PMID: 20464741.

5. Hansen MS, Kjær ML, Andersen J. Efficacy of Plug Treatment for Complex Anorectal Fistulae: Long-term Danish Results. Ann Coloproctol. 2019;35(3):123-8. Epub 20190320. doi: 10.3393/ac.2018.07.14. PubMed PMID: 30889947; PubMed Central PMCID: PMCPMC6625774.

6. Y S, H L, SY C, HL T, YY W, D Z. Clinical Observation of Multi-Incision Drainage Procedure for the Treatment of High Horseshoe Anal Fistula. Journal of Shanghai University of Traditional Chinese Medicine. 2021;35(05):32-6. doi: 10.16306/j.1008-861x.2021.05.006.

7. Z X. Clinical Observation of Simultaneous Multi-Incision Seton Drainage Procedure for the Treatment of High Complex Anal Fistulas in 58 Cases. Sichuan Medical Journal. 2012;33(09):1606-7. doi: 10.16252/j.cnki.issn1004-0501-2012.09.046.

8. Vogel JD, Johnson EK, Morris AM, Paquette IM, Saclarides TJ, Feingold DL, et al. Clinical Practice Guideline for the Management of Anorectal Abscess, Fistula-in-Ano, and Rectovaginal Fistula. Dis Colon Rectum. 2016;59(12):1117-33. doi: 10.1097/DCR.0000000000000733. PubMed PMID: 27824697.

9. Y S. Clinical Observation of Multi-Incision Seton Drainage Procedure for the Treatment of High Horseshoe Anal Fistula [master]: Shanghai University of Traditional Chinese Medicine; 2020.

10. Pastar I, Stojadinovic O, Yin NC, Ramirez H, Nusbaum AG, Sawaya A, et al. Epithelialization in Wound Healing: A Comprehensive Review. Adv Wound Care (New Rochelle). 2014;3(7):445-64. doi: 10.1089/wound.2013.0473. PubMed PMID: 25032064; PubMed Central PMCID: PMCPMC4086220.

11. Wang TH, Kiu KT, Yen MH, Chang TC. Comparison of the short-term outcomes of using DST and PPH staplers in the treatment of grade III and IV hemorrhoids. Sci Rep. 2020;10(1):5189. Epub 20200323. doi: 10.1038/s41598-020-62141-5. PubMed PMID: 32251336; PubMed Central PMCID: PMCPMC7089945.

12. Eichhorn W, Haase M, Kluwe L, Zeuch J, Smeets R, Hanken H, et al. Increased Postoperative Bleeding Risk among Patients with Local Flap Surgery under Continued Clopidogrel Therapy. Biomed Res Int. 2015;2015:120903. Epub 20150806. doi: 10.1155/2015/120903. PubMed PMID: 26345612; PubMed Central PMCID: PMCPMC4543372.

13. El-Matary W, Walters TD, Huynh HQ, deBruyn J, Mack DR, Jacobson K, et al. Higher Postinduction Infliximab Serum Trough Levels Are Associated With Healing of Fistulizing Perianal Crohn's Disease in Children. Inflamm Bowel Dis. 2019;25(1):150-5. doi: 10.1093/ibd/izy217. PubMed PMID: 29912413; PubMed Central PMCID: PMCPMC6290776.

14. Mei Z, Feng Q, Du P, Li B, Fang C, Gu J, et al. Surgical treatment for cryptoglandular and Crohn's perianal fistulas: Protocol of an umbrella review. PLoS One. 2021;16(5):e0251460. Epub

20210513. doi: 10.1371/journal.pone.0251460. PubMed PMID: 33984014; PubMed Central PMCID: PMCPMC8118242.

15. Mei Z, Li Y, Zhang Z, Zhou H, Liu S, Han Y, et al. Development of screening tools to predict the risk of recurrence and related complications following analfistula surgery: protocol for a prospective cohort study. BMJ Open. 2020;10(3):e035134. Epub 20200304. doi: 10.1136/bmjopen-2019-035134. PubMed PMID: 32139494; PubMed Central PMCID: PMCPMC7059513.
