# Supplementary material for: Evaluating the efficacy of multi-incision and tube-dragging therapy combined with laser closure for high horseshoe-shaped anal fistula: Protocol of a prospective, randomized, controlled trial
Source: PLoS One. 2024 Sep 27;19(9):e0307653. doi: 10.1371/journal.pone.0307653 (PMC11432866; doi:10.1371/journal.pone.0307653)
Supplement: S3 File — (PDF) [file pone.0307653.s003.pdf]

多切口拖管联合激光闭合治疗高位马蹄形肛瘘的前瞻性、随机对照试验方案

主要研究者：郑德，上海中医药大学附属曙光医院 肛肠科

电子邮件：zd1232@sina.com

地址：上海市浦东新区张衡路528号

版本：1.0

日期：2021年11月2日

摘要

引文:

高位马蹄形肛瘘 (HHAF) 是一种复杂且具有挑战性的疾病，在治疗中存在相当大的困难。我们目前正在研究一种新型手术技术，该技术结合了多切口拖管疗法以及激光闭合来治疗 HHAF。由于目前缺乏评估这种方法的严格证据，因此必须进行一项精心设计的随机对照临床试验，以比较这种新方法与切开挂线术的有效性和安全性差异。

方法与分析:

本研究为一项前瞻性、随机、对照、干预性试验，旨在严谨比较两种不同治疗方法的效果。经过初步筛选，共纳入64名符合标准的成年门诊患者，并随机分为 MITD-LaC 组和对照组，每组各32人。两组分别接受 MITD-LaC 治疗与切开挂线疗法治疗。本试验设计的核心目的在于对两种治疗方法进行稳健的比较分析。主要观察指标为伤口愈合时间，以评估不同治疗方式的疗效差异。此外，试验还设置了多个次要终点，包括术后1、3、5天的肛门疼痛程度（采用视觉模拟量表进行量化评估）、术后30天内的大便失禁情况（通过克利夫兰诊所佛罗里达失禁评分进行标准化测量）、术后1个月内术后并发症的发生情况，以及术后6个月内患者的生活质量状况（运用肛瘘患者生活质量问卷评分进行综合评价）。通过本研究的开展，我们期望能够为临床治疗提供更为准确、科学的依据，为改善患者生活质量贡献力量。

讨论：

本研究作为首个针对MITD-LaC短期成效展开的随机对照试验，旨在提供高质量、可靠的临床数据支持，以指导临床实践的有效进行。此外，本研究还结合了主观与客观指标的综合结果评估方法，力求全面、准确地反映MITD-LaC 在 HHAF 治疗中的实际表现。鉴于这种多维度的评估方式，MITD-LaC 在治疗HHAF方面展现出了更广泛的潜在应用前景。因此，通过科学、严谨的临床试验手段，获取更为明确、权威的证据，对于进一步验证并推广这种治疗方法具有重要意义。

研究方案

1. 研究背景

肛瘘作为一种常见的非恶性肛门直肠疾病，其特征在于直肠或肛管与肛周皮肤之间形成异常通道，常表现为肛周不适、脓性分泌物及瘙痒等症状。其中，高位马蹄形肛瘘（HHAF）作为一种特殊的深后肛瘘类型，其病灶延伸至坐骨直肠间隙，形成一侧或两侧的马蹄形通道，因此其临床治疗颇具挑战性[1,2]。由于HHAF位置深在、管道结构复杂，且涉及肛门括约肌等重要结构，导致其治疗难度加大[3-5]。

目前，我们的研究焦点在于验证一种结合多切口拖管疗法与激光闭合技术的外科手术方案在高位马蹄形肛瘘治疗中的有效性。该手术方案以多切口拖管疗法（MITD）为基础，通过放置多个引流管替代传统开放切口，旨在最大程度地保护肛门括约肌功能的同时，确保伤口充分引流。此外，术后还结合了拖拉、冲洗和抽吸等措施，以加速坏死组织的清除，促进伤口愈合。

与切开挂线术（ITD）相比，多切口拖管疗法已证实能够减少术后并发症、缩短愈合时间，并有效保护肛门括约肌功能[6,7]。而MITD-LaC方法中激光闭合技术的引入，则进一步提升了手术效果。激光闭合技术能够精确消除瘘管壁上的炎性上皮和坏死组织，具有减少创伤、促进恢复以及减轻术后疼痛等多重优势。

然而，目前关于MITD-LaC的疗效尚未经过科学严谨的随机对照试验进行全面评估。因此，我们有必要开展一项随机对照试验，以比较MITD-LaC与切开挂线疗法在治疗高位马蹄形肛瘘方面的疗效差异，为临床决策提供更为科学、可靠的依据。

2. 研究对象

在入组之前，每位患者均需经过磁共振成像（MRI）以及经直肠超声（TRUS）两项检查的严格筛选，以精确界定HHAF病变的边界范围及其与毗邻组织的具体关系。关于HHAF的诊断工作，将由至少两名具备丰富经验的高级放射学专家共同进行，并互相确认诊断结果的准确性。

关于入组标准，我们明确规定了以下几点：首先，入组患者需为年龄在18至65周岁之间的男性或女性住院患者，并确诊为HHAF；其次，患者所患肛瘘类型需为完全性马蹄形肛瘘或半马蹄形肛瘘。此外，对于相关诊断标准的制定，我们还将参照美国结肠直肠外科医师协会（ASCRS）所发布的《肛门直肠脓肿、肛瘘和直肠阴道瘘管理临床实践指南（2016）》[8]，以确保诊断的严谨性与科学性。

至于排除标准，我们亦制定了以下详细规定：其一，年龄不满18周岁或超过65周岁的患者将被排除在外；其二，孕妇或哺乳期妇女亦不符合入组条件；其三，经过电子肠镜检查证实患有恶性肿瘤的患者将被排除；其四，患有特定感染疾病（如结核病、克罗恩病、艾滋病毒感染等）的患者同样不符合入组要求；其五，患有混合痔、肛裂、肛周湿疹等严重肛周疾病的患者亦将被排除；其六，存在认知障碍或无法理解和提供知情同意的患者亦不在入组之列；最后，同时参与另一项临床试验的患者亦将被排除。

3. 研究设计

本研究是一项前瞻性、随机、对照、干预性的临床试验，旨在探讨不同手术治疗方法对HHAF患者的疗效。本研究将共纳入64例HHAF患者，并严格按照随机原则将其分为MITD-LaC组和ITD组。在研究过程中，我

们将全面收集患者的相关临床资料，并进行深入的前瞻性分析，以期能够为HHAF患者的手术治疗提供更为科学、合理的依据。

4. 样本量估计

本研究样本量的确定是基于两个样本均值比较的统计学原理。据先前研究报告指出，采用切开线疗治疗HHAF的患者的平均愈合时间为62.69 ± 3.54天[9]。在本项研究中，我们预设MITD-LaC 组的平均愈合时间将缩短3天，并将允许误差(δ)设定为3天，已知的标准差为3.54天。依据统计表，我们设定本研究的显著性水平为α=0.05（即I型错误率），并确定β=0.10（即Ⅱ型错误率），由此得出$Z_{\alpha} = 1.960$ 和 $Z_{\beta} = 1.282$。经过精确计算，每组至少需要纳入25名参与者，此结果系采用功效分析和样本量程序2021（PASS, NCSS Statistical Software, Kaysville, UT, USA）计算得出。鉴于我们预计的20%退出率，我们计划为两组共招募64名参与者，并据此将32名参与者分配给每组。随机化过程将采用SAS软件生成的随机数表进行，该随机数表将根据病例总数制定。治疗分配将依据序列号Q01至Q64进行，从而生成一个全面的随机代码表。这些序列号将依据门诊就诊顺序依次分配至各参与者。

5. 干预措施

（1）MITD-LaC术式

在肛瘘远端处做一个约1.5cm的切口，分离皮下组织，暴露瘘管。顺着瘘管的走向，小心地将瘘管解剖至肛后缘，然后做放射状切口。对于完全性HHAF，将执行相同的操作，将解剖延伸至对侧肛缘，直至到达内口。随后，切除瘘管，并仔细清除周围所有炎性坏死组织。将位于齿线上方的瘘管切开，将光纤导丝放入瘘管顶端，然后激光汽化和消融瘘管。在止血并进行彻底的伤口冲洗后，将橡胶引流管小心地插入相邻切口之间并牢固固定。橡胶引流管壁上应穿孔多个小孔，每个小孔直径约为2mm，以优化引流效率。

（2）ITD术式

切除或剥离齿状线以下瘘管及脓腔，清创口缘，确保瘘管内炎性坏死组织全部摘除，同时彻底清除外口周围纤维化管壁及瘢痕组织。在内口处肛缘做长约3cm放射状切口，切开、刮除瘘管，清除坏死组织，彻底切除内口及感染性病变。伤口开放引流后，将探针插入内口，经肛门引出，在探针末端系橡皮圈，拉出肛门，将橡皮圈两端拉紧，丝线固定。修剪创缘，常规压迫止血、填塞。随后，将进行标准包扎和固定程序，以固定伤口区域。

6. 临床观察指标

（1）基线信息

手术前将记录两组患者的基线数据信息（如性别、年龄、身高、体重、初发/复发等）。

（2）主要结局指标

主要结局指标是伤口愈合时间，定义为从手术当天到手术伤口完全上皮化的时间[10]。伤口愈合情况将通过医生在患者术后常规门诊随访中的局部检查和综合评估来确认。

（3）次要结局指标

次要结局指标包括使用视觉模拟量表 (VAS) 评估术后第 1、3 和 5 天的疼痛程度。此外，将使用克利夫兰诊所佛罗里达失禁评分 (CCF-IS) 评估术后 30 天内的大便失禁评分。还将观察术后并发症，例如感染、尿潴留、出血、伤口愈合延迟和术后一个月内复发。此外，将使用肛瘘患者生活质量问卷评分 (QoLAF-QS) 评估患者术后六个月的生活质量。

术后感染的发生定义为手术部位出现明显疼痛，伴有肿胀和脓疱，伴或不伴有发烧，需要进行抗生素治疗。术后尿潴留定义为在膀胱过度膨胀的情况下无法自行排尿，需要导尿以缓解[11]。术后出血是指需要紧急处理的过度出血，如出血部位局部按压止血[12]。伤口愈合延迟是指任何伤口在 3 个月内未完全重新上皮化[13]。复发是指在之前的手术部位周围再次出现瘘管，通常需要使用 TRUS 或 EMRI 进行诊断辅助[14, 15]。

参考文献

1. Pezim ME. Successful treatment of horseshoe fistula requires deroofing of deep postanal space. Am J Surg. 1994;167(5):513-5. doi: 10.1016/0002-9610(94)90247-x. PubMed PMID: 8185039.
2. Usui A, Ishiyama G, Nishio A, Kawamura M, Kono Y, Ishiyama Y. Two-Stage Complete Deroofing Fistulotomy Approach for Horseshoe Fistula: Successful Surgery Leaving Continence Intact. Ann Coloproctol. 2021;37(3):153-8. Epub 20210112. doi: 10.3393/ac.2020.06.08. PubMed PMID: 33445836; PubMed Central PMCID: PMCPMC8273714.
3. Browder LK, Sweet S, Kaiser AM. Modified Hanley procedure for management of complex horseshoe fistulae. Tech Coloproctol. 2009;13(4):301-6. Epub 20091008. doi: 10.1007/s10151-009-0539-6. PubMed PMID: 19813077.
4. Jacob TJ, Perakath B, Keighley MR. Surgical intervention for anorectal fistula. Cochrane Database Syst Rev. 2010;(5):Cd006319. Epub 20100512. doi: 10.1002/14651858.CD006319.pub2. PubMed PMID: 20464741.
5. Hansen MS, Kjær ML, Andersen J. Efficacy of Plug Treatment for Complex Anorectal Fistulae: Long-term Danish Results. Ann Coloproctol. 2019;35(3):123-8. Epub 20190320. doi: 10.3393/ac.2018.07.14. PubMed PMID: 30889947; PubMed Central PMCID: PMCPMC6625774.
6. 邵雨, 陆宏, 陈诗雨, 汤慧丽, 王奕韵, 郑德. 多切口拖管术治疗高位马蹄型肛瘘的临床观察. 上海中医药大学学报. 2021;35(05):32-6. doi: 10.16306/j.1008-861x.2021.05.006.
7. 徐征. 同期多切口切开挂线引流术治疗高位复杂性肛瘘58例临床观察. 四川医学. 2012;33(09):1606-7. doi: 10.16252/j.cnki.issn1004-0501-2012.09.046.
8. Vogel JD, Johnson EK, Morris AM, Paquette IM, Saclarides TJ, Feingold DL, Steele SR. Clinical Practice Guideline for the Management of Anorectal Abscess, Fistula-in-Ano, and Rectovaginal Fistula. Dis Colon Rectum. 2016;59(12):1117-33. doi: 10.1097/dcr.0000000000000733. PubMed PMID: 27824697.
9. 邵雨. 多切口拖管术治疗高位马蹄型肛瘘的临床观察 [硕士]2020.
10. Pastar I, Stojadinovic O, Yin NC, Ramirez H, Nusbaum AG, Sawaya A, et al. Epithelialization in Wound Healing: A Comprehensive Review. Adv Wound Care (New Rochelle). 2014;3(7):445-64. doi: 10.1089/wound.2013.0473. PubMed PMID: 25032064; PubMed Central PMCID: PMCPMC4086220.
11. Wang TH, Kiu KT, Yen MH, Chang TC. Comparison of the short-term outcomes of using DST and PPH staplers in the treatment of grade III and IV hemorrhoids. Sci Rep. 2020;10(1):5189. Epub 20200323. doi: 10.1038/s41598-020-62141-5. PubMed PMID: 32251336; PubMed Central PMCID: PMCPMC7089945.
12. Eichhorn W, Haase M, Kluwe L, Zeuch J, Smeets R, Hanken H, et al. Increased Postoperative Bleeding Risk among Patients with Local Flap Surgery under Continued Clopidogrel Therapy. Biomed Res Int. 2015;2015:120903. Epub 20150806. doi: 10.1155/2015/120903. PubMed PMID: 26345612; PubMed Central PMCID: PMCPMC4543372.
13. El-Matary W, Walters TD, Huynh HQ, deBruyn J, Mack DR, Jacobson K, et al. Higher Postinduction Infliximab Serum Trough Levels Are Associated With Healing of Fistulizing Perianal Crohn's Disease in Children. Inflamm Bowel Dis. 2019;25(1):150-5. doi: 10.1093/ibd/izy217. PubMed PMID: 29912413; PubMed Central PMCID: PMCPMC6290776.
14. Mei Z, Feng Q, Du P, Li B, Fang C, Gu J, et al. Surgical treatment for cryptoglandular and Crohn's perianal fistulas: Protocol of an umbrella review. PLoS One. 2021;16(5):e0251460. Epub 20210513. doi: 10.1371/journal.pone.0251460. PubMed PMID: 33984014; PubMed Central PMCID: PMCPMC8118242.
15. Mei Z, Li Y, Zhang Z, Zhou H, Liu S, Han Y, et al. Development of screening tools to predict the risk of recurrence and related complications following anal fistula surgery: protocol for a prospective cohort study. BMJ Open. 2020;10(3):e035134. Epub 20200304. doi: 10.1136/bmjopen-2019-035134. PubMed PMID: 32139494; PubMed Central PMCID: PMCPMC7059513.
